# Assessing Prototype Immunoassays for Plasma GFAP on the LUMIPULSE Platform

**DOI:** 10.1002/alz.094628

**Published:** 2025-01-09

**Authors:** Hisashi Nojima

**Affiliations:** ^1^ FUJIREBIO IINC., Hachioji, Tokyo Japan

## Abstract

**Background:**

There is increasing evidence that the pathogenesis of Alzheimer’s disease (AD) involves various immunological responses. Indeed, astrocyte reactivity or astrocytosis is a pathological process that is commonly found surrounding amyloid‐β plaques in the brains of AD patients. Although the exact role of astrocytosis is still unrevealed, monitoring astrocyte activity using biomarkers leads to a better understanding of the underlying mechanism of AD pathogenesis. Glial fibrillary acidic protein (GFAP) is commonly used as a reactive astrocyte biomarker following injury or stress in the central nervous system. Here, we developed an assay for plasma GFAP on the LUMIPULSE *G* platform, and evaluated the preliminary performance.

**Method:**

The LUMIPULSE *G* System is a fully automated chemiluminescent enzyme immunoassay (CLEIA) platform that processes samples in ready‐to‐use immunoreaction cartridges within 30 minutes. The assay employs a pair of in‐house developed monoclonal antibodies to detect GFAP. Analytical assay characteristics including sensitivity, precision and correlation between paired serum and plasma samples were assessed. The analytical studies comprised a series of buffer, EDTA plasma and serum samples, each tested in multiple replicates. Preliminary clinical performance was investigated using a small set of commercially‐ available samples.

**Result:**

All tested analytical parameters met our acceptance criteria. Healthy control samples from a young age group exhibited results above the limit of quantification (LoQ). The LoQ was determined based on the assessment of low concentrated plasma GFAP samples. Imprecision was less than 10% CV for buffer‐based as well as plasma samples. There was a statistically significant correlation between plasma and serum GFAP levels (r = 0.99, *p* <0.0001). ROC curve analysis showed the greatest AUC for plasma GFAP to distingish AD patients from healthy controls.

**Conclusion:**

This preliminary evaluation of the Lumipulse *G* GFAP Plasma prototype assay showed promising results with regards to high sensitivity, low variability, and clinical utility. Further studies with well‐characterised and diverse cohorts are needed to validate the assay’s clinical performance.

